# The use of oral anticoagulation at the time of acute COVID-19 infection and subsequent development of long-COVID/post-acute sequelae of SARS-CoV-2 infection

**DOI:** 10.1007/s11239-025-03096-0

**Published:** 2025-04-05

**Authors:** Freddy Frost, José Miguel Rivera-Caravaca, Gregory Y. H. Lip

**Affiliations:** 1 Liverpool Centre for Cardiovascular Science, University of Liverpool, Liverpool John Moores University and Liverpool Heart & Chest Hospital, Liverpool, UK; 2 Faculty of Nursing, University of Murcia, Instituto Murciano de Investigación Biosanitaria (IMIB- Arrixaca), CIBERCV, Murcia, Spain; 3 Danish Center for Health Services Research, Department of Clinical Medicine, Aalborg University, Aalborg, Denmark

## Abstract

Long COVID (LC) or post-acute sequelae of SARS-CoV-2 infection (PASC) is defined as ongoing, relapsing or new symptoms/conditions persisting after an acute COVID-19 infection. Given the potential role of oral anticoagulants (OAC) in treating thrombotic sequelae of LC/PASC, we investigated whether prevalent OAC use at the time of acute COVID-19 infection was associated with reduced development of LC/PASC. Retrospective cohort study within the TriNetx network. The primary cohort was defined as adults with a confirmed diagnosis of COVID-19. We defined OAC users as those who had received OACs (either direct-acting OACs [DOACs] or vitamin K antagonists [VKA]) in the preceding 3-months and non-users as those without OAC use within the previous 12-months. The primary outcome was a composite of 9 features associated with LC/PASC We identified 38,409 DOAC users, 19,243 VKA users, and 2,329,771 non-OAC users with acute COVID-19 infection. After successful propensity score matching (PSM), we found an increased risk of LC/PASC features in those receiving DOAC compared to non-OAC (HR [95% CI] 1.50 [1.35 to 1.68], *p* < 0.0001), and in VKA users compared to non-OACs (HR [95% CI] 1.98 [1.78 to 2.20], *p* < 0.0001), while DOAC users were at reduced risk compared to VKA users (HR [95% CI] 0.71 [0.62 to 0.81], *p* < 0.0001). We found no evidence that prevalent OAC at the time of acute COVID-19 infection was associated with reduced risk of LC/PASC. Further work is needed to understand whether there is a role for OAC therapy in the management of LC/PASC.

**Supplementary Information:**

The online version contains supplementary material available at 10.1007/s11239-025-03096-0.

## Introduction

Long COVID (LC) or post-acute sequelae of SARS-CoV-2 infection (PASC) is defined as ongoing, relapsing or new symptoms/conditions persisting after an acute COVID-19 infection and is a major public health concern. At present, there are no available treatments, and little is known about the pathophysiology although persistent thrombotic abnormalities and microangiopathy have been linked with poor outcomes from acute COVID-19 as well as LC/PASC. Given the putative role of oral anticoagulants (OAC) (e.g. direct-acting oral anticoagulants [DOACs] and vitamin K antagonists [VKA]) in the treatment of such thrombotic sequelae, we investigated whether prevalent OAC use, i.e. existing use of OAC at the time of acute COVID-19 infection was associated with reduced development of LC/PASC.

## Methods

### Data source

We undertook a retrospective cohort study within the TriNetX network, a global federated health research network with access to electronic medical records (EMRs) from academic and community hospitals mainly located in the United States (U.S.). The primary cohort was defined as adults (18 years or older) with a confirmed diagnosis of COVID-19 (ICD-10 U07.1) between January 2020 and January 2023. We defined OAC users as those who had received OACs in the preceding three months and non-users as those without OAC use within the previous 12 months. Index was considered the date of COVID-19 diagnosis.

### Outcomes

Primary outcome of interest was a composite of 9 features associated with LC/PASC as previously described [1]. The constituent clinical features were as follows:

- Cognitive dysfunction.
- Myalgia.
- Headache.
- Pain.
- Anxiety/depression.
- Abdominal symptoms.
- Abnormal breathing.
- Chest/throat pain.

### Statistical analyses

Propensity score matching (PSM) 1:1, was used to control for differences between cohorts. Cohort matching was performed as previously described [1]. Any baseline characteristic with a standardised mean difference < 0.100 was considered well matched. After PSM, Cox-regression proportional hazard models were used to calculate hazard ratios (HRs) with 95% confidence intervals (CIs) for the risk of PASC between groups. All analyses were performed in the TriNetX platform which incorporates R (v4.3.1, R Foundation for Statistical Foundation, Vienna, Austria).

## Results

We identified 38,409 DOAC users, 19,243 VKA users and 2,329,771 non-OAC users with acute COVID-19 infection within the TriNetX network. Mean (SD) age in the three groups was 70.3 (14.1), 67.8 (15.5) and 49.4 (19.0) years, respectively (Table 1, Supplementary Tables 1 and 2).

**Table 1:** Characteristics pre and post propensity score matching for DOAC users and VKA users

Code	Characteristic	Pre-PSM					Post-PSM				
		DOAC		VKA		SMD	DOAC		VKA		SMD
		*n* or mean	% or SD	*n* or mean	% or SD		*n* or mean	% or SD	*n* or mean	% or SD	
AI	Age at Index	70.3	14.1	67.8	15.5	0.174	68.6	15.1	68.3	15.1	0.023
2186-5	Not Hispanic or Latino	28,841	75.1%	14,406	74.9%	0.005	14,343	75.9%	14,187	75.1%	0.019
2106-3	White	27,670	72.0%	13,476	70.0%	0.044	13,494	71.4%	13,338	70.6%	0.018
M	Male	19,650	51.2%	10,646	55.3%	0.084	10,430	55.2%	10,406	55.1%	0.003
UN	Unknown Ethnicity	7789	20.3%	3765	19.6%	0.018	3605	19.1%	3715	19.7%	0.015
2054-5	Black or African American	5991	15.6%	3171	16.5%	0.024	3034	16.1%	3091	16.4%	0.008
2131-1	Unknown Race	3732	9.7%	2144	11.1%	0.047	1968	10.4%	2021	10.7%	0.009
2135-2	Hispanic or Latino	1779	4.6%	1072	5.6%	0.043	948	5.0%	994	5.3%	0.011
2028-9	Asian	757	2.0%	300	1.6%	0.031	282	1.5%	297	1.6%	0.006
I30-I52	Other forms of heart disease (deprecated 2021)	23,738	61.8%	9580	49.8%	0.244	9417	49.8%	9556	50.6%	0.015
I10-I16	Hypertensive diseases	23,354	60.8%	9443	49.1%	0.237	9195	48.7%	9392	49.7%	0.021
E11	Type 2 diabetes mellitus	11,683	30.4%	4890	25.4%	0.112	4731	25.0%	4870	25.8%	0.017
N17-N19	Acute kidney failure and chronic kidney disease	11,088	28.9%	4892	25.4%	0.078	4631	24.5%	4828	25.6%	0.024
I20-I25	Ischemic heart diseases	11,347	29.5%	4464	23.2%	0.144	4389	23.2%	4454	23.6%	0.008
C00-D49	Neoplasms	8638	22.5%	3041	15.8%	0.171	3083	16.3%	3030	16.0%	0.008
J40-J47	Chronic lower respiratory diseases	8781	22.9%	3175	16.5%	0.161	3074	16.3%	3164	16.7%	0.013
E66	Overweight and obesity	7548	19.7%	3228	16.8%	0.075	3050	16.1%	3195	16.9%	0.021
F30-F39	Mood [affective] disorders	6419	16.7%	2445	12.7%	0.113	2328	12.3%	2424	12.8%	0.015
F40-F48	Anxiety, dissociative, stress-related, somatoform and other nonpsychotic mental disorders	6009	15.6%	2164	11.2%	0.129	2099	11.1%	2148	11.4%	0.008
J44	Other chronic obstructive pulmonary disease	5652	14.7%	1954	10.2%	0.139	1931	10.2%	1953	10.3%	0.004
F10-F19	Mental and behavioral disorders due to psychoactive substance use	3881	10.1%	1237	6.4%	0.134	1233	6.5%	1235	6.5%	0.000
J45	Asthma	3027	7.9%	1152	6.0%	0.075	1079	5.7%	1144	6.1%	0.015
I63	Cerebral infarction	2312	6.0%	931	4.8%	0.052	855	4.5%	917	4.9%	0.016
F17.2	Nicotine dependence	2664	6.9%	859	4.5%	0.107	838	4.4%	858	4.5%	0.005
D80-D89	Certain disorders involving the immune mechanism	1849	4.8%	766	4.0%	0.041	722	3.8%	748	4.0%	0.007
K76	Other diseases of liver	1740	4.5%	684	3.6%	0.050	630	3.3%	673	3.6%	0.012
J43	Emphysema	1594	4.2%	455	2.4%	0.101	471	2.5%	455	2.4%	0.005
F03	Unspecified dementia	1410	3.7%	426	2.2%	0.086	419	2.2%	426	2.3%	0.003
C81-C96	Malignant neoplasms of lymphoid, hematopoietic and related tissue	1492	3.9%	372	1.9%	0.116	412	2.2%	371	2.0%	0.015
M06	Other rheumatoid arthritis	951	2.5%	353	1.8%	0.044	360	1.9%	350	1.9%	0.004
K74	Fibrosis and cirrhosis of liver	649	1.7%	346	1.8%	0.008	314	1.7%	335	1.8%	0.009
K76.0	Fatty (change of) liver, not elsewhere classified	850	2.2%	316	1.6%	0.042	303	1.6%	314	1.7%	0.005
M32	Systemic lupus erythematosus (SLE)	353	0.9%	344	1.8%	0.075	282	1.5%	267	1.4%	0.007
E10	Type 1 diabetes mellitus	680	1.8%	294	1.5%	0.019	253	1.3%	291	1.5%	0.017
F02	Dementia in other diseases classified elsewhere	662	1.7%	210	1.1%	0.054	202	1.1%	209	1.1%	0.004
J40	Bronchitis, not specified as acute or chronic	453	1.2%	178	0.9%	0.025	186	1.0%	177	0.9%	0.005
K76.8	Other specified diseases of liver	530	1.4%	176	0.9%	0.044	168	0.9%	174	0.9%	0.003
G30	Alzheimer’s disease	527	1.4%	146	0.8%	0.060	153	0.8%	146	0.8%	0.004
J47	Bronchiectasis	331	0.9%	113	0.6%	0.032	120	0.6%	112	0.6%	0.005
F01	Vascular dementia	399	1.0%	113	0.6%	0.050	111	0.6%	113	0.6%	0.001
K72	Hepatic failure, not elsewhere classified	196	0.5%	119	0.6%	0.014	101	0.5%	109	0.6%	0.006
Z59	Problems related to housing and economic circumstances	286	0.7%	80	0.4%	0.043	92	0.5%	80	0.4%	0.009
J42	Unspecified chronic bronchitis	284	0.7%	84	0.4%	0.040	83	0.4%	84	0.4%	0.001
J41	Simple and mucopurulent chronic bronchitis	263	0.7%	66	0.3%	0.048	75	0.4%	66	0.3%	0.008

Abbreviations: DOAC = Direct oral anticoagulant; PSM = Propensity score matching; VKA = vitamin K antagonist; SMD = Standardised mean difference; SD = Standard deviation

Overall, in the 6 months following an acute COVID-19 infection, unadjusted incidence of composite LC/PASC was 32.5% in DOAC users, 32.3% in VKA users, and 10.9% in non-OAC users.

**Fig. 1:**
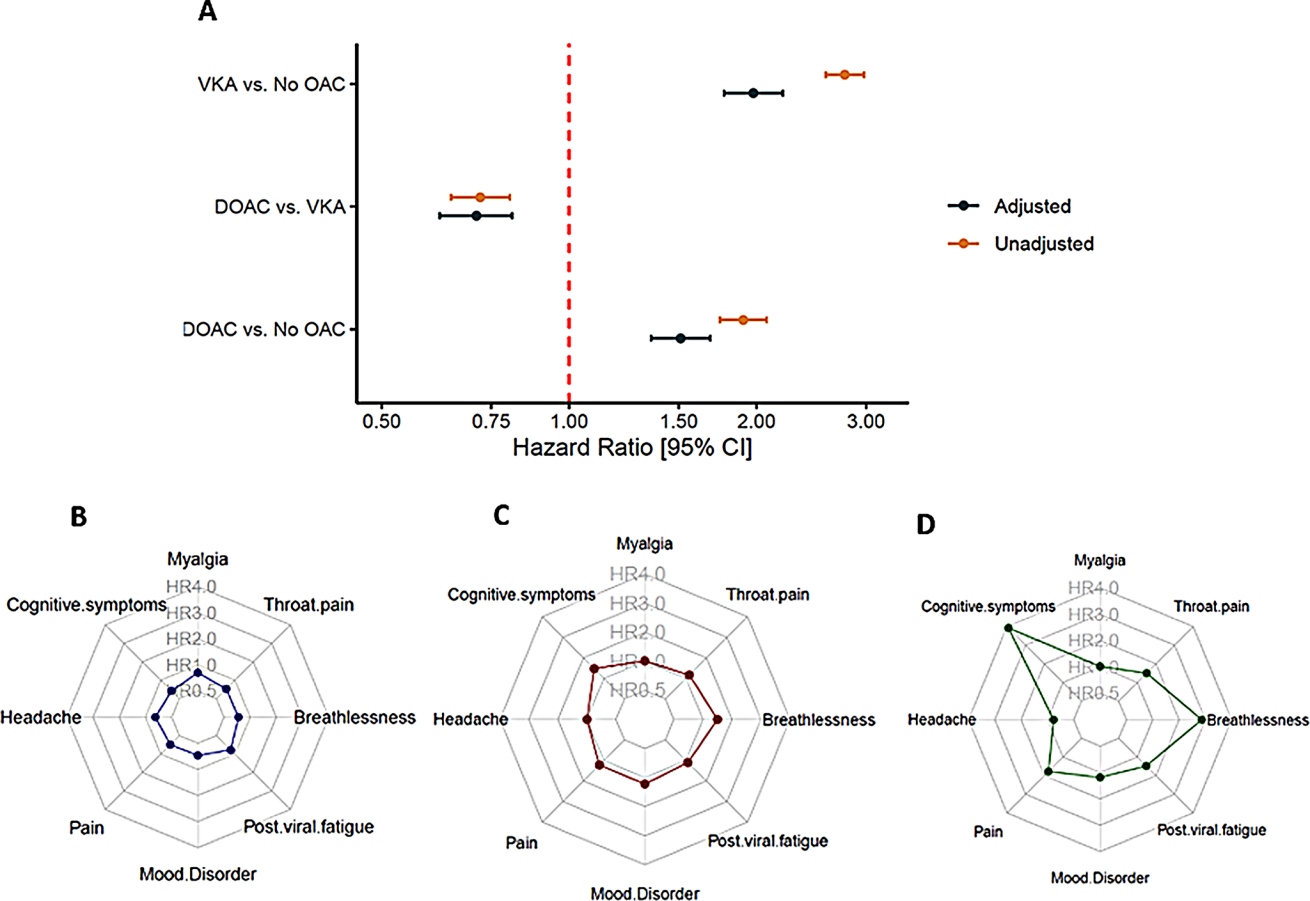
Forest plot of Hazard Ratio and 95% confidence interval for composite outcome (**A**); Radar plots showing comparative risk of individual LC/PASC features 6 months after acute COVID-19 infection between DOAC users and VKA users (**B**); DOAC and non-OAC users (**C**); VKA users and non-OAC users (**D**). Abbreviations: DOAC = direct-acting oral anticoagulant; VKA = vitamin K antagonist

After successful PSM, we found an increased risk of LC/PASC features in those receiving DOAC compared to non-OAC (HR [95% CI] 1.50 [1.35 to 1.68], *p* < 0.0001), and in VKA users compared to non-OACs (HR [95% CI] 1.98 [1.78 to 2.20], *p* < 0.0001), while DOAC users were at reduced risk compared to VKA users (HR [95% CI] 0.71 [0.62 to 0.81], *p* < 0.0001), see Fig. 1A.

For all groups, breathlessness, post viral fatigue and cognitive symptoms were the most frequent new features (data not shown). The between-group risk for each feature is presented in Fig. 1B, C and D.

## Discussion

In this study, we investigated the risk of LC/PASC in people already receiving OACs at the time of acute COVID-19 infection. Our main finding is that OAC use does not appear to be associated with any reduction in LC/PASC symptom development and, in general, OAC users were associated with increased risk of LC/PASC.

To our knowledge, this is the first study to investigate the potential for prevalent OAC use at the time of COVID-19 infection to modulate the risk of LC/PASC. Acute COVID-19 infection is recognised a major risk factor for acute thrombotic complications including both arterial and venous thromboembolic disease and sustained prothrombotic changes have been reported long after resolution of acute infection [2, 3]. Thrombotic disease has been implicated in the pathophysiology of LC/PASC in a number of studies, for example Pasini *et al.* described persistence of altered D-Dimer profiles in people living with LC/PASC and *Pretorius et al.* described persistent microclot deposits in people living with LC/PASC [4]. These findings have led the putative suggestion of long-term intense anticoagulation as a treatment for LC/PASC however to date this approach has not been tested in clinical trials [5]. 

Our findings of no benefit for prevalent OAC use on PASC should not be interpreted as evidence for a lack of effect of OAC for PASC. For example, a mechanistic clotting study found extremely high resistance of LC/PASC microclots to fibrinolysis and the same study group have suggested intense anticoagulation incorporating dual antiplatelet therapy alongside OAC may be indicated on that basis. We found insufficient people receiving such regimens within the TriNetX platform to conduct analyses, likely due to the established bleeding risk associated with this therapeutic combination [6]. 

Another unresolved question, not covered by our study, is the potential role of OAC when a diagnosis of LC/PASC is already made. Following COVID-19, chronic inflammation may persist and contribute to endothelial cell activation, platelet stimulation, and increased inflammatory responses. This process can upregulate procoagulant factors and impair the protective function of the vascular endothelium, leading to abnormal coagulation [7]. Under such conditions, starting OAC -or maintaining this therapy if already prescribed- may lead to lower risk of worse clinical outcomes [8, 9]. However, it should be noted that the overall incidence of vascular thromboembolic events after hospital discharge for COVID-19 remains low (approximately 2%) [10]. What kind of anticoagulation could be employed in this clinical context required further investigation. Hence, our results should be taken to confirm the urgent need for robust data from randomised clinical trials to determine the role for DOACs in the management of LC/PASC associated microclots. Importantly, since COVID-19 is an endothelial problem, it could be hypothesized that if infection quickly overcomes tenuous anticoagulation triggering a cytokine storm and leading to excessive inflammation, the underlying systemic/tissue hypercoagulability conditions of patients requiring previous OAC would quickly synergize with SARS-CoV-2, increasing the likelihood LC/PASC.

There are limitations to consider for this study. First, the data were gathered from healthcare organizations EMRs, potentially leading to underreporting of certain health conditions. Thus, despite we accounted for well-known confounders, residual confounding may still exist due to unmeasured variables. TriNetX does not represent territory wide healthcare systems so follow-up, presentation and recording of some LC/PASC outcomes could have theoretically occurred but not been captured here, potentially leading to underestimation of LC/PASC. Similarly, TriNetX is enriched with secondary care institutions meaning COVID-19 infections may be unrepresentative in terms of severity. We are unable to distinguish dose, and we therefore are unable to assess impact of DOAC dosage on outcomes. There is no agreed definition of the constituent components of a long-covid or LC/PASC diagnosis, and although we aligned our analyses with previously reported algorithms, our results may be confounded by heterogenous reporting of conditions.

In conclusion, we found no evidence prevalent OAC at the time of acute COVID-19 infection is associated with reduced risk of LC/PASC. However, patients on OAC have an enhanced pro-inflammatory and pro-thrombotic state and are more fragile and vulnerable. Therefore, OAC could have played a protective role in this high-risk population, limiting the appearance of more severe LC/PASC. Further work is needed to understand whether there is a role for OAC therapy in the management of LC/PASC.

## Electronic supplementary material

Below is the link to the electronic supplementary material.

Supplementary Material 1

## Data Availability

No datasets were generated or analysed during the current study.
