# Supplementary material for: The use of oral anticoagulation at the time of acute COVID-19 infection and subsequent development of long-COVID/post-acute sequelae of SARS-CoV-2 infection
Source: J Thromb Thrombolysis. 2025 Apr 5;58(4):585–9. doi: 10.1007/s11239-025-03096-0 (PMC12043746; doi:10.1007/s11239-025-03096-0)
Supplement: Supplementary file 1 — Supplementary Material 1 [file 11239_2025_3096_MOESM1_ESM.docx]

**Supplementary Material**

**Supplementary Table 1.** Characteristics pre and post propensity score matching for DOAC users and non-OAC users.

| **Code** | **Characteristic** | **Pre-PSM** | | | |  | **Post-PSM** | | | |  |
| --- | --- | --- | --- | --- | --- | --- | --- | --- | --- | --- | --- |
|  |  | **DOAC** | | **Non-OAC** | | **SMD** | **DOAC** | | **Non-OAC** |  | **SMD** |
|  |  | **n or mean** | **% or SD** | **n or mean** | **% or SD** |  | **n or mean** | **% or SD** | **n or mean** | **% or SD** |  |
| AI | **Age at Index** | 70.3 | 14.1 | 49.4 | 19.0 | 1.2 | 70.3 | 14.1 | 71.0 | 13.6 | 0.045 |
| 2186-5 | **Not Hispanic or Latino** | 28841 | 75.09% | 1432544 | 61.49% | 0.295 | 28838 | 75.09% | 28826 | 75.06% | 0.001 |
| 2106-3 | **White** | 27670 | 72.04% | 1455467 | 62.47% | 0.205 | 27669 | 72.04% | 27946 | 72.77% | 0.016 |
| M | **Male** | 19650 | 51.16% | 965915 | 41.46% | 0.195 | 19648 | 51.16% | 19704 | 51.30% | 0.003 |
| UN | **Unknown Ethnicity** | 7789 | 20.28% | 668982 | 28.71% | 0.197 | 7789 | 20.28% | 7852 | 20.45% | 0.004 |
| 2054-5 | **Black or African American** | 5991 | 15.60% | 330947 | 14.21% | 0.039 | 5989 | 15.59% | 5878 | 15.31% | 0.008 |
| 2131-1 | **Unknown Race** | 3732 | 9.72% | 443256 | 19.03% | 0.268 | 3732 | 9.72% | 3627 | 9.44% | 0.009 |
| 2135-2 | **Hispanic or Latino** | 1779 | 4.63% | 228245 | 9.80% | 0.201 | 1779 | 4.63% | 1728 | 4.50% | 0.006 |
| 2028-9 | **Asian** | 757 | 1.97% | 80020 | 3.44% | 0.090 | 757 | 1.97% | 749 | 1.95% | 0.002 |
| I30-I52 | **Other forms of heart disease (deprecated 2021)** | 23738 | 61.80% | 159993 | 6.87% | 1.418 | 23735 | 61.80% | 11698 | 30.46% | 0.662 |
| I10-I16 | **Hypertensive diseases** | 23354 | 60.80% | 427074 | 18.33% | 0.964 | 23351 | 60.80% | 23531 | 61.27% | 0.010 |
| E11 | **Type 2 diabetes mellitus** | 11683 | 30.42% | 202572 | 8.70% | 0.569 | 11680 | 30.41% | 11692 | 30.44% | 0.001 |
| I20-I25 | **Ischemic heart diseases** | 11347 | 29.54% | 100488 | 4.31% | 0.714 | 11344 | 29.54% | 10820 | 28.17% | 0.030 |
| N17-N19 | **Acute kidney failure and chronic kidney disease** | 11088 | 28.87% | 103987 | 4.46% | 0.693 | 11085 | 28.86% | 10624 | 27.66% | 0.027 |
| J40-J47 | **Chronic lower respiratory diseases** | 8781 | 22.86% | 162584 | 6.98% | 0.457 | 8778 | 22.86% | 8280 | 21.56% | 0.031 |
| C00-D49 | **Neoplasms** | 8638 | 22.49% | 178449 | 7.66% | 0.424 | 8636 | 22.49% | 8724 | 22.72% | 0.005 |
| E66 | **Overweight and obesity** | 7548 | 19.65% | 160331 | 6.88% | 0.383 | 7546 | 19.65% | 7238 | 18.85% | 0.020 |
| F30-F39 | **Mood [affective] disorders** | 6419 | 16.71% | 170011 | 7.30% | 0.293 | 6419 | 16.71% | 6118 | 15.93% | 0.021 |
| F40-F48 | **Anxiety, dissociative, stress-related, somatoform and other nonpsychotic mental disorders** | 6009 | 15.65% | 207270 | 8.90% | 0.207 | 6009 | 15.65% | 5774 | 15.03% | 0.017 |
| J44 | **Other chronic obstructive pulmonary disease** | 5652 | 14.72% | 50043 | 2.15% | 0.464 | 5649 | 14.71% | 5125 | 13.34% | 0.039 |
| F10-F19 | **Mental and behavioral disorders due to psychoactive substance use** | 3881 | 10.10% | 100300 | 4.31% | 0.226 | 3880 | 10.10% | 3660 | 9.53% | 0.019 |
| J45 | **Asthma** | 3027 | 7.88% | 100463 | 4.31% | 0.150 | 3027 | 7.88% | 2848 | 7.42% | 0.018 |
| F17.2 | **Nicotine dependence** | 2664 | 6.94% | 71968 | 3.09% | 0.177 | 2663 | 6.93% | 2523 | 6.57% | 0.015 |
| I63 | **Cerebral infarction** | 2312 | 6.02% | 17001 | 0.73% | 0.296 | 2311 | 6.02% | 1989 | 5.18% | 0.036 |
| D80-D89 | **Certain disorders involving the immune mechanism** | 1849 | 4.81% | 27473 | 1.18% | 0.214 | 1848 | 4.81% | 1751 | 4.56% | 0.012 |
| K76 | **Other diseases of liver** | 1740 | 4.53% | 39271 | 1.69% | 0.164 | 1740 | 4.53% | 1453 | 3.78% | 0.037 |
| J43 | **Emphysema** | 1594 | 4.15% | 15369 | 0.66% | 0.229 | 1594 | 4.15% | 1387 | 3.61% | 0.028 |
| C81-C96 | **Malignant neoplasms of lymphoid, hematopoietic and related tissue** | 1492 | 3.89% | 18900 | 0.81% | 0.204 | 1490 | 3.88% | 1410 | 3.67% | 0.011 |
| F03 | **Unspecified dementia** | 1410 | 3.67% | 13815 | 0.59% | 0.214 | 1410 | 3.67% | 1324 | 3.45% | 0.012 |
| M06 | **Other rheumatoid arthritis** | 951 | 2.48% | 16403 | 0.70% | 0.142 | 951 | 2.48% | 849 | 2.21% | 0.018 |
| K76.0 | **Fatty (change of) liver, not elsewhere classified** | 850 | 2.21% | 25555 | 1.10% | 0.088 | 850 | 2.21% | 800 | 2.08% | 0.009 |
| E10 | **Type 1 diabetes mellitus** | 680 | 1.77% | 15630 | 0.67% | 0.100 | 679 | 1.77% | 670 | 1.75% | 0.002 |
| F02 | **Dementia in other diseases classified elsewhere** | 662 | 1.72% | 8746 | 0.38% | 0.133 | 662 | 1.72% | 600 | 1.56% | 0.013 |
| K74 | **Fibrosis and cirrhosis of liver** | 649 | 1.69% | 12334 | 0.53% | 0.111 | 649 | 1.69% | 587 | 1.53% | 0.013 |
| K76.8 | **Other specified diseases of liver** | 530 | 1.38% | 10037 | 0.43% | 0.100 | 530 | 1.38% | 432 | 1.13% | 0.023 |
| G30 | **Alzheimer's disease** | 527 | 1.37% | 7027 | 0.30% | 0.118 | 527 | 1.37% | 525 | 1.37% | 0.000 |
| J40 | **Bronchitis, not specified as acute or chronic** | 453 | 1.18% | 12363 | 0.53% | 0.071 | 453 | 1.18% | 468 | 1.22% | 0.004 |
| F01 | **Vascular dementia** | 399 | 1.04% | 3554 | 0.15% | 0.115 | 399 | 1.04% | 338 | 0.88% | 0.016 |
| M32 | **Systemic lupus erythematosus (SLE)** | 353 | 0.92% | 7332 | 0.32% | 0.077 | 353 | 0.92% | 336 | 0.88% | 0.005 |
| J47 | **Bronchiectasis** | 331 | 0.86% | 4603 | 0.20% | 0.092 | 331 | 0.86% | 302 | 0.79% | 0.008 |
| Z59 | **Problems related to housing and economic circumstances** | 286 | 0.75% | 6253 | 0.27% | 0.067 | 286 | 0.75% | 249 | 0.65% | 0.012 |
| J42 | **Unspecified chronic bronchitis** | 284 | 0.74% | 2725 | 0.12% | 0.095 | 284 | 0.74% | 223 | 0.58% | 0.020 |
| J41 | **Simple and mucopurulent chronic bronchitis** | 263 | 0.69% | 2529 | 0.11% | 0.092 | 263 | 0.69% | 208 | 0.54% | 0.018 |
| K72 | **Hepatic failure, not elsewhere classified** | 196 | 0.51% | 3831 | 0.16% | 0.060 | 196 | 0.51% | 178 | 0.46% | 0.007 |

Abbreviations: DOAC=Direct oral anticoagulant; PSM=Propensity score matching; VKA=vitamin K antagonist; SMD=Standardised mean difference; SD=Standard deviation

**Supplementary Table 2.** Characteristics pre and post propensity score matching for VKA users and non-OAC users.

| **Code** | **Characteristic** | **Pre-PSM** | | | |  | **Post-PSM** | | | |  |
| --- | --- | --- | --- | --- | --- | --- | --- | --- | --- | --- | --- |
|  |  | **VKA** | | **Non-OAC** | | **SMD** | **VKA** | | **Non-OAC** |  | **SMD** |
|  |  | **n or mean** | **% or SD** | **n or mean** | **% or SD** |  | **n or mean** | **% or SD** | **n or mean** | **% or SD** |  |
| AI | **Age at Index** | 67.8 | 15.5 | 49.4 | 19.0 | 1.058 | 67.8 | 15.5 | 68.6 | 15.1 | 0.058 |
| 2186-5 | **Not Hispanic or Latino** | 14406 | 74.9% | 1432544 | 61.5% | 0.290 | 14405 | 74.9% | 14357 | 74.6% | 0.006 |
| 2106-3 | **White** | 13476 | 70.0% | 1455467 | 62.5% | 0.160 | 13475 | 70.0% | 13412 | 69.7% | 0.007 |
| M | **Male** | 10646 | 55.3% | 965915 | 41.5% | 0.280 | 10646 | 55.3% | 10673 | 55.5% | 0.003 |
| UN | **Unknown Ethnicity** | 3765 | 19.6% | 668982 | 28.7% | 0.215 | 3765 | 19.6% | 3810 | 19.8% | 0.006 |
| 2054-5 | **Black or African American** | 3171 | 16.5% | 330947 | 14.2% | 0.063 | 3171 | 16.5% | 3276 | 17.0% | 0.015 |
| 2131-1 | **Unknown Race** | 2144 | 11.1% | 443256 | 19.0% | 0.222 | 2144 | 11.1% | 2155 | 11.2% | 0.002 |
| 2135-2 | **Hispanic or Latino** | 1072 | 5.6% | 228245 | 9.8% | 0.159 | 1072 | 5.6% | 1075 | 5.6% | 0.001 |
| 2028-9 | **Asian** | 300 | 1.6% | 80020 | 3.4% | 0.120 | 300 | 1.6% | 278 | 1.4% | 0.009 |
| I30-I52 | **Other forms of heart disease (deprecated 2021)** | 9580 | 49.8% | 159993 | 6.9% | 1.083 | 9579 | 49.8% | 9381 | 48.8% | 0.021 |
| I10-I16 | **Hypertensive diseases** | 9443 | 49.1% | 427074 | 18.3% | 0.688 | 9442 | 49.1% | 9199 | 47.8% | 0.025 |
| N17-N19 | **Acute kidney failure and chronic kidney disease** | 4892 | 25.4% | 103987 | 4.5% | 0.615 | 4891 | 25.4% | 4525 | 23.5% | 0.044 |
| E11 | **Type 2 diabetes mellitus** | 4890 | 25.4% | 202572 | 8.7% | 0.456 | 4889 | 25.4% | 4775 | 24.8% | 0.014 |
| I20-I25 | **Ischemic heart diseases** | 4464 | 23.2% | 100488 | 4.3% | 0.570 | 4463 | 23.2% | 4205 | 21.9% | 0.032 |
| E66 | **Overweight and obesity** | 3228 | 16.8% | 160331 | 6.9% | 0.310 | 3228 | 16.8% | 3001 | 15.6% | 0.032 |
| J40-J47 | **Chronic lower respiratory diseases** | 3175 | 16.5% | 162584 | 7.0% | 0.299 | 3174 | 16.5% | 2944 | 15.3% | 0.033 |
| C00-D49 | **Neoplasms** | 3041 | 15.8% | 178449 | 7.7% | 0.255 | 3041 | 15.8% | 2939 | 15.3% | 0.015 |
| F30-F39 | **Mood [affective] disorders** | 2445 | 12.7% | 170011 | 7.3% | 0.181 | 2445 | 12.7% | 2232 | 11.6% | 0.034 |
| F40-F48 | **Anxiety, dissociative, stress-related, somatoform and other nonpsychotic mental disorders** | 2164 | 11.2% | 207270 | 8.9% | 0.078 | 2164 | 11.2% | 1960 | 10.2% | 0.034 |
| J44 | **Other chronic obstructive pulmonary disease** | 1954 | 10.2% | 50043 | 2.1% | 0.338 | 1953 | 10.2% | 1760 | 9.1% | 0.034 |
| F10-F19 | **Mental and behavioral disorders due to psychoactive substance use** | 1237 | 6.4% | 100300 | 4.3% | 0.094 | 1236 | 6.4% | 1156 | 6.0% | 0.017 |
| J45 | **Asthma** | 1152 | 6.0% | 100463 | 4.3% | 0.076 | 1152 | 6.0% | 1052 | 5.5% | 0.022 |
| I63 | **Cerebral infarction** | 931 | 4.8% | 17001 | 0.7% | 0.252 | 930 | 4.8% | 780 | 4.1% | 0.038 |
| F17.2 | **Nicotine dependence** | 859 | 4.5% | 71968 | 3.1% | 0.072 | 859 | 4.5% | 830 | 4.3% | 0.007 |
| D80-D89 | **Certain disorders involving the immune mechanism** | 766 | 4.0% | 27473 | 1.2% | 0.177 | 766 | 4.0% | 660 | 3.4% | 0.029 |
| K76 | **Other diseases of liver** | 684 | 3.6% | 39271 | 1.7% | 0.117 | 684 | 3.6% | 620 | 3.2% | 0.018 |
| J43 | **Emphysema** | 455 | 2.4% | 15369 | 0.7% | 0.140 | 455 | 2.4% | 420 | 2.2% | 0.012 |
| F03 | **Unspecified dementia** | 426 | 2.2% | 13815 | 0.6% | 0.138 | 426 | 2.2% | 360 | 1.9% | 0.024 |
| C81-C96 | **Malignant neoplasms of lymphoid, hematopoietic and related tissue** | 372 | 1.9% | 18900 | 0.8% | 0.097 | 372 | 1.9% | 341 | 1.8% | 0.012 |
| M06 | **Other rheumatoid arthritis** | 353 | 1.8% | 16403 | 0.7% | 0.101 | 353 | 1.8% | 302 | 1.6% | 0.020 |
| K74 | **Fibrosis and cirrhosis of liver** | 346 | 1.8% | 12334 | 0.5% | 0.118 | 346 | 1.8% | 336 | 1.7% | 0.004 |
| M32 | **Systemic lupus erythematosus (SLE)** | 344 | 1.8% | 7332 | 0.3% | 0.145 | 343 | 1.8% | 272 | 1.4% | 0.029 |
| K76.0 | **Fatty (change of) liver, not elsewhere classified** | 316 | 1.6% | 25555 | 1.1% | 0.047 | 316 | 1.6% | 275 | 1.4% | 0.017 |
| E10 | **Type 1 diabetes mellitus** | 294 | 1.5% | 15630 | 0.7% | 0.082 | 294 | 1.5% | 279 | 1.5% | 0.006 |
| F02 | **Dementia in other diseases classified elsewhere** | 210 | 1.1% | 8746 | 0.4% | 0.084 | 210 | 1.1% | 181 | 0.9% | 0.015 |
| J40 | **Bronchitis, not specified as acute or chronic** | 178 | 0.9% | 12363 | 0.5% | 0.046 | 178 | 0.9% | 153 | 0.8% | 0.014 |
| K76.8 | **Other specified diseases of liver** | 176 | 0.9% | 10037 | 0.4% | 0.059 | 176 | 0.9% | 165 | 0.9% | 0.006 |
| G30 | **Alzheimer's disease** | 146 | 0.8% | 7027 | 0.3% | 0.063 | 146 | 0.8% | 135 | 0.7% | 0.007 |
| K72 | **Hepatic failure, not elsewhere classified** | 119 | 0.6% | 3831 | 0.2% | 0.073 | 119 | 0.6% | 122 | 0.6% | 0.002 |
| J47 | **Bronchiectasis** | 113 | 0.6% | 4603 | 0.2% | 0.062 | 113 | 0.6% | 111 | 0.6% | 0.001 |
| F01 | **Vascular dementia** | 113 | 0.6% | 3554 | 0.2% | 0.072 | 113 | 0.6% | 84 | 0.4% | 0.021 |
| J42 | **Unspecified chronic bronchitis** | 84 | 0.4% | 2725 | 0.1% | 0.061 | 84 | 0.4% | 58 | 0.3% | 0.022 |
| Z59 | **Problems related to housing and economic circumstances** | 80 | 0.4% | 6253 | 0.3% | 0.025 | 80 | 0.4% | 84 | 0.4% | 0.003 |
| J41 | **Simple and mucopurulent chronic bronchitis** | 66 | 0.3% | 2529 | 0.11% | 0.049 | 66 | 0.3% | 54 | 0.3% | 0.000 |

Abbreviations: DOAC=Direct oral anticoagulant; PSM=Propensity score matching; VKA=vitamin K antagonist; SMD=Standardised mean difference; SD=Standard deviation
